# Butyrate and Lauric Acid Attenuate Amyloid‐Beta‐Induced Oxidative, Mitochondrial, and Lipidomic Dysregulation in Human iPSC‐Derived Astrocytes and Neurons

**DOI:** 10.1002/alz70861_108390

**Published:** 2025-12-23

**Authors:** RM. Uththara Sachinthanie Senarath, Lotta E Oikari, Prashant Bharadwaj, Vijay Jayasena, Luke Whiley, Monique Ryan, Ralph N Martins, Warnakulasuriya Mary Ann Dipika Binosha Fernando

**Affiliations:** ^1^ Edith Cowan University, Perth, Western Australia Australia; ^2^ Alzheimer's Research Australia, Perth, Western Australia Australia; ^3^ QIMR Berghofer Medical Research Institute, Herston, QLD Australia; ^4^ Western Sydney University, Sydney, NSW Australia; ^5^ Australian National Phenome Centre, Health Futures Institute, Murdoch University, Murdoch, Western Australia Australia; ^6^ Lions Alzheimer's Foundation, Perth, Western Australia Australia; ^7^ Department of Biomedical Sciences, Macquarie University, Macquarie Park, NSW Australia; ^8^ Alzheimer’s Research Australia, Perth, Western Australia Australia

## Abstract

**Background:**

In Alzheimer’s disease (AD), astrocytes undergo reactive changes that can exert both protective and detrimental effects on neurons, thereby influencing neuronal survival and contributing to disease progression. Increasing attention has been directed toward elucidating the mechanisms underlying astrocyte dysfunction and neuroinflammation to identify novel therapeutic targets. Butyrate—a short‐chain fatty acid produced by gut microbiota—has demonstrated anti‐inflammatory and neuroprotective properties. Similarly, lauric acid (LA), a medium‐chain fatty acid, has shown potential in attenuating amyloid‐beta (Aβ)‐induced neurotoxicity, enhancing mitochondrial function, and modulating neuronal excitability. Despite these findings, the mechanisms by which they modulate astrocyte and neuronal function remain poorly understood. This study investigates the effects of butyrate and LA on oxidative stress, mitochondrial dysfunction, and lipidomic alterations in human‐induced pluripotent stem cells (iPSCs)‐derived astrocytes, neurons, and spontaneous cultures.

**Method:**

Astrocytes, neurons, and spontaneous co‐cultures were differentiated from iPSCs derived from a healthy donor. Cultures were treated with synthetic Aβ (20 µM), sodium butyrate (NaB) (100 µM), or LA (100 µM), alone and in combination. Oxidative stress, mitochondrial dysfunction and the lipidomic changes of these cells was measured using Quantitative PCR, Catalase activity, Mitochondrial ToxGlo™ Assay and Liquid Chromatography‐Mass Spectrometry (LC‐MS) respectively.

**Result:**

Aβ exposure significantly suppressed catalase expression in astrocytes, indicating elevated oxidative stress. Treatment with NaB and LA exhibit partial restoration of catalase expression, with the most effects observed in mixed cultures, suggesting enhanced cellular resilience. Furthermore, expression of Mitofusin‐1, a key regulator of mitochondrial fusion, was downregulated following Aβ treatment. Functional assays revealed butyrate and LA enhance antioxidant enzyme activity and improved mitochondrial integrity over time, with astrocytes and mixed cultures showing the most pronounced protective effect. The lipid profiles indicate that Aβ induces greater lipid variation in neurons compared to astrocytes and spontaneous cultures.

**Conclusion:**

This study highlights the modulatory roles of butyrate and lauric acid on oxidative stress pathways, mitochondrial dynamics, and lipidomic profiles in iPSC‐derived brain cell cultures subjected to amyloid‐beta toxicity. These results support further exploration of butyrate and LA as therapeutic agents targeting neuroinflammation and metabolic dysregulation in AD.

